# From traditional robotic deployments towards assisted robotic deployments in nuclear decommissioning

**DOI:** 10.3389/frobt.2025.1432845

**Published:** 2025-02-18

**Authors:** Erwin Jose Lopez Pulgarin, Dave Hopper, Jon Montgomerie, James Kell, Joaquin Carrasco, Guido Herrmann, Alexander Lanzon, Barry Lennox

**Affiliations:** ^1^ Department of Electrical and Electronics Engineering, The University of Manchester, Manchester, United Kingdom; ^2^ Amentum, Warrington, United Kingdom

**Keywords:** teleoperation, robotics, robot deployment, haptic digital twin, semi-autonomy, training

## Abstract

The history around teleoperation and deployment of robotic systems in constrained and dangerous environments such as nuclear is a long and successful one. From the 1940s, robotic manipulators have been used to manipulate dangerous substances and enable work in environments either too dangerous or impossible to be operated by human operators. Through the decades, technical and scientific advances have improved the capabilities of these devices, whilst allowing for more tasks to be performed. In the case of nuclear decommissioning, using such devices for remote inspection and remote handling has become the only solution to work and survey some areas. Such applications deal with challenging environments due to space constrains, lack of up-to-date structural knowledge of the environment and poor visibility, requiring much training and planning to succeed. There is a growing need to speed these deployment processes and to increase the number of decommissioning activities whilst maintaining high levels of safety and performance. Considering the large number of research and innovation being done around improving robotic capabilities, numerous potential benefits could be made by translating them to the nuclear decommissioning use cases. We believe such innovations, in particular improved feedback mechanisms from the environment during training and deployments (i.e., Haptic Digital Twins) and higher modes of assisted or supervised control (i.e., Semi-autonomous operation) can play a large role. We list some of the best practices currently being followed in the industry around teleoperation and robotic deployments and the potential benefits of implementing the aforementioned innovations.

## 1 Introduction

The use of robots in the nuclear industry has a long and rich history (Bogue, 2011), going from teleoperated serial manipulator mechanisms to some of the latest research in the use of Remotely Operated Vehicles (ROV) and mobile robots (Tsitsimpelis et al., 2019) for robotic inspection. Most devices referred to as robots for the nuclear industry are used to perform tasks where human presence is either limited or not possible due to environmental factors such as radioactive hazards. Some of their uses happen in different stages of a reactor’s lifecycle, including its commissioning and construction, during maintenance operations, waste disposal services and during its decommissioning. The benefits of such technologies are still being explored, and are maturing into products ready for deployments in nuclear sites such as Sellafield (United Kiingdom) (Sellafield, 2023) and Fukushima Daiichi (Japan) (Tugal et al., 2022; Zhang et al., 2025).

Beyond mechanical manipulators and exploratory vehicles, other technologies and robots could help with tasks that are still being performed manually during any of the reactor’s lifecycle stages. Maintenance and decommissioning tasks are a clear example, as they involve expert human operators performing tasks whilst wearing protective outfits or using gloveboxes to manipulate dangerous substances in an isolated environment. The use of different robots such as teleoperated robotic manipulators for decommissioning can bring safety and operational benefits, such as for operations currently being performed inside gloveboxes (Tokatli et al., 2021). However, many challenges remain for widespread adoption of these tools, and particularly to reach the task performance levels that an expert human operator can achieve (Pulgarin et al., 2022). Regardless of the growing need to have larger capacity to perform tasks at a faster pace, the demanding requirements around safety and performance (i.e., repeatability, accuracy), slow down and increase costs of producing tested and certifiable platforms regardless of the technology.

The term digital twin has gained popularity in the recent decade (Cryer et al., 2023), as it encompasses many technologies used to sense, store, display and manipulate information related to a remote asset (Cryer et al., 2023). Such systems are designed to mimic the remote assets, and tend to include simulation and visualization capabilities that allow for greater control of the process (Tu et al., 2023; Cryer et al., 2023). Feedback from and towards the digital twin can come from different modalities (e.g., visual, sound), being haptics and/or force (Elsner et al., 2022) one with large potential and interest. The creation of Haptic Digital Twins (HDT) that allow for realistic representations of remote sites, either as offline mock-ups or online representations, could be used during training and deployments in the context of nuclear decommissioning.

The large interest around increased levels of autonomy (i.e., robots adapting to environmental changes to fulfil a goal with little to no human input) enables further opportunities for imbuing robots with the capabilities to operate in constrained and complex environments. A popular example of autonomy in highly complex systems is the one for autonomous vehicles (Wang et al., 2020), where parts of the driving task are performed by the vehicle itself with only supervisory input from the driver. In the context of remotely controlling or interacting with a robot, any assistance provided that eases the control of the robot or improves any operational performance metric (Li et al., 2023a) [i.e., Assisted Operations (AO)] can prove beneficial. Such levels of autonomy and assistance require human input (Li et al., 2023b; Pruks and Ryu, 2022) for all cases where full autonomy is not possible, which for the current state-of-the-art includes many industrial use cases. The use of Assisted Operations for teleoperated robotic control could improve task performance to expert operator-level without exposing operators to hazardous environments.

**FIGURE 1:**
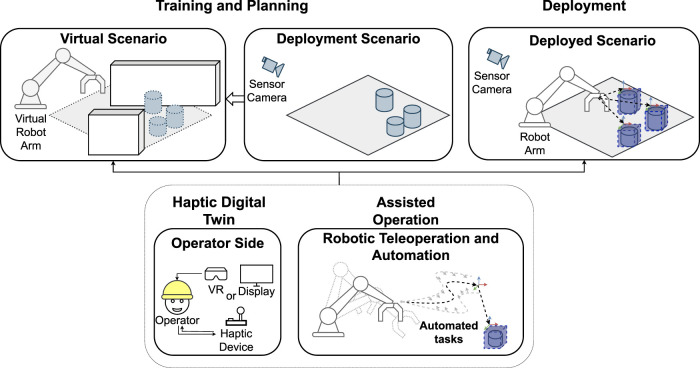
High-level depiction of Haptic Digital Twins (HDT) and Assisted Operations (AO) thought different stages of a robotic deployment.

This perspective paper introduces our approach to achieve Assisted Robotic Deployments. We strongly believe that improved multimodal feedback mechanisms [i.e., Haptic Digital Twins (HDT)] and adaptive modes of assisted or supervised control [i.e., Assisted Operation (AO)] can play a large role in improving nuclear decommissioning operations. HDTs can be used as training and deployment platforms, replicating real-world deployment scenarios with realistic haptic-enabled visualizations; AOs are used to automate tasks to be performed in the deployment environment and improve teleoperation. A visual description of these can be seen in Figure 1. Such Assisted Robotic Deployments would produce safe and performing deployments, increasing capacity and reducing deployment times. The remaining of the paper is organized as follows: Sections 2.1, 2.2 include the technical challenges behind deploying robotic devices for nuclear decommissioning and best practices in the industry. Section 2.3 builds on the previously mentioned technical challenges to discuss how HDT and AO can play a role in improving overall operation. Section 3 discusses current limitations and research aims.

## 2 Robotic deployments in the nuclear industry

### 2.1 Complexities of robotic deployments in nuclear decommissioning

The use of remote handling systems, particularly robotic ones, is beneficial to nuclear decommissioning in several ways. Primarily, robotics enables to perform tasks that simply may not be possible with any other means, since the target may be in a location too difficult or hazardous to reach with manned access. An additional significant benefit is the reduction in exposure to harmful radiation to operators who might have had to enter an environment if robotic alternatives did not exist.

Regardless of its potential benefits, the application of robotic systems in this environment is not simple. There are considerable challenges to overcome, both physical and regulatory, for any deployment to be successful. Arguably the biggest issue facing the system, and particularly for any operator, is the nature of the environment. Knowledge about the environment is crucial to scope the hardware to be deployed, and to perform all necessary planning. It is common that historical information in the form of design documentation and historical records is incomplete or not up to date. Often the challenge is confirming that what is inside a remote cell is what is expected – and it is very common that this is not the case. This uncertainty inevitably leads to additional work required to overcome the gaps in knowledge. Some studies suggest (Brotherhood et al., 2022) that the level of uncertainty in remote cells can reach up to 300% when comparing preliminary decommissioning plans and the actual work required.

To understand and agree on the concept of operations for a decommissioning exercise, the most up-to-date knowledge of the target area is required. Therefore, the first step is to perform an initial survey of the area to acquire data that will fill in the gaps in knowledge. If attempting to decommission using a robotic system, this might suggest the use of a specific robot that is equipped with a range of scanners. Such scanners include visual cameras, thermal cameras, chemical analysis, radiation sensors, LiDAR, and photogrammetry for geometric shape reconstruction. The acquisition of these data sources – by whatever means – and subsequent compilation onto a single existence or map of the environment can provide the operator with the information required to start planning a decommissioning task. This plan will then lead to the collation of requirements and ultimately a specification of a robotic system that will meet the decommissioning goals of the site owner. Given the unstructured nature of the decommissioning target, it is very unlikely that off the shelf robotic systems will be able to meet the specification. This may lead to the customisation of a system or possibly a bespoke design and build to meet the needs.

It should not be understated that the skill and experience of the team behind the design and deployment of such a robotic solution for nuclear decommissioning is of utmost importance. The ability to draw from prior examples of interventions that went well — and crucially, what to avoid — is of significant benefit to all involved and often is the difference between a successful application or otherwise. These operators can make sense of multiple, often suboptimal data sources such as poor-quality video feeds, or having to control multiple manipulators simultaneously and still completing the task required in the majority of instances.

### 2.2 Best practices for robotic deployments in nuclear decommissioning

Considering the previous statement around how challenging the environment is during nuclear decommissioning, any strategy around robotic deployment should limit time spent inside the cell, but particularly ensure benign and retrievable systems. This can be explained as ensuring that during deployment, the decommissioning task does not become more difficult to perform, or additional waste is not produced in the form of a robotic system that cannot be retrieved. Rule of thump concepts such as “do not make the situation worse” and “do not get stuck” apply during any deployment, regardless of its complexity.

Professional teams take advantage of various formal tools to ensure the successful application of robotics in nuclear environments, such as:

• The Hazard and Operability process: The HazOp process is a structured and systematic examination of a complex system, such as a site listed for decommissioning. This process enables the user to identify hazards to personnel, equipment, or the environment, as well as operability issues that may affect the operations efficiency – with a primary focus on safety. The International Electrotechnical Commission (IEC) published an application guide (Standard, 2001) that provides a framework for operation, and the application of this process can provide a good start to planning any activity.
• Systems Engineering: This provides a structured set of desk-based tools for the collection and understanding of requirements and subsequent specifications for devices to be built and used. It provides a way of ensuring that all interdisciplinary stakeholders are engaged and aware of the intentions of a robotic application.
• Physical mock-ups: These mock-ups are an effective way of providing a safe test area for the training and development of a robotic system. They can also help to describe or explain both the concept of operation and the safety case to the asset owner and the regulatory body. The obvious benefit to physical mock-ups is the additional views that an operator could be provided, which would not be available in the real application, but are nevertheless of use for development purposes.
• Fault Tree Analysis: This is a type of failure analysis that examines the possibilities of what might result from an undesirable fault. This is a structured approach to understanding the logic leading up to a failure. From this analysis, mitigation strategies can be put in place to either prevent the failure from occurring, or at least to reduce the impact of such a failure taking place. In all instances, the design needs to adhere to As Low as Reasonably Possible (ALARP) principles (Hurst et al., 2019), which involves weighing a risk against the trouble, time, and money needed to control it.
• Safety Case: A safety case (ONR, 2020) should include all documentation that demonstrates high standards of nuclear safety and radioactive waste management to satisfy both the asset owner and the regulatory body. It facilitates relevant discussions, as it captures useful information for the design and deployment of robotic systems.

### 2.3 Assisted training and modes of operation for deployments

Digital twins (Cryer et al., 2023), and in particular Haptic Digital Twins (HDT) can have a large role in providing assistance in robotic deployment at both training and remote deployment stages. An HDT to assist remote deployments would integrate live sensor data from different sources, such as 3D cameras and radiation sensors, for its visualization (see Figure 1). It would integrate a physics-based robot simulations with kinematic constraints replicating and allowing to predict the robot whilst interacting with the environment (Tu et al., 2023). It would provide the capability to remotely control the robot and to provide haptic feedback (Pruks and Ryu, 2022) related to the state of the robot and its operation (e.g., robot motion and collisions with the environment). Such HDTs would take advantage of modern computer graphics technologies such as Neural Radiance Fields (NeRF) (Tancik et al., 2023) and Gaussian Splatting (Kerbl et al., 2023); these allow to use visual and spatial information (e.g., 2D from cameras and 3D point clouds) to create 3D representations of spaces with high-definition visualizations, including its surface information (Yu et al., 2022) and the reconstructed geometry (Millane et al., 2024). Due to the availability of sensors that produce visual and spatial information with capabilities suited for different lightings and operation conditions (e.g., Global Shutter, Polarizer, stereo or time-of-flight depth perception) obtaining high-quality visual and spatial information is achievable. Modern computing with dedicated Graphical Computing Units (GPU), high-speed sensor interfaces and optimized physic engines for robotics are needed to make use of NeRF and haptic rendering techniques for visuo-haptic rendering. Visually rich, haptic-enabled representations of a real decommissioning environment would make the HDT into a useful tool for robotic deployment activities.

There are benefits of using HDTs for training and deployment beyond having a more complete and realistic user interface (Tugal et al., 2023). An HDT could enhance the current practice of using physical mock-ups for extensive operator training under a specific deployment plan. Operators require training in a representative and realistic environment, using the same or similar robotic system to be deployed (i.e., Graphical User Interface, local control device and remote robot). However, there are usually only one or two sets of the robotic system to deploy, as these systems are costly and time-consuming to commission. By using HDTs for training operators, we can use the same interface designed for deployments and training, whilst creating realistic environments without the need for physical mock-ups.

Creating Assisted Operations (AO) to improve task performance (i.e., any metrics of success that measures task completion and quality) can be realised in many ways, based on the level of autonomy given to the remote robot. Initial levels of assisted operation include constrained Cartesian motion and velocity compensation, which allow for motion per axis or motion on a plane defined in space, either whilst holding or changing the orientation of the robot’s end-effector. Additional levels of assistance would include automatic collision avoidance between all the parts of the remote robot and the environment, removing the robot from dangerous configurations whilst providing useful feedback to the user about the robot’s motion. Automated actions would add another level of assistance, by including pre-programmed motions (e.g., open entry hatch, pick-up tool, return to initial position, scan glovebox floor) or, sequences of motions starting from a relative position (e.g., move around a specific geometry or object in the scene, bring objects to a desired position) (see Figure 1); such actions would aid during teleoperation when complete sensor feedback from the environment is not possible, which is a common occurrence in a constrained and hazardous environment such as a glovebox. Using AOs would expand the current practice of using Fault Tree Analysis, by explicitly avoiding faulty states or state combinations (e.g., collisions), and would help create the Safety Case by automated testing of the low risk achieved in the fault analysis.

A more advanced level of AOs would integrate all previous assistance modes, allowing for manual or automatic switch between assistance modes and full manual teleoperation. Such levels of assistance are called shared control in the literature, acting at either the control level of the robot or at the user input and feedback level [i.e., haptic guidance (Li et al., 2023a)]. Achieving these levels of assistance requires sensor feedback from the robot and its environment, similar to the one needed for HDTs. However, the main challenge lies in dynamically computing safe trajectories and control policies to handle object grasping, tool handling and other tasks, considering an operator controlling or supervising the whole operation. This is still under research, earning it different names, including semi-autonomy, shared autonomy, and shared teleoperation among others (Elsner et al., 2022; Pruks and Ryu, 2022; Li et al., 2023b). The final objective of any AOs should be to either improve task performance directly with better control capabilities, or to positively influence this metric by providing more intuitive and configurable capabilities to the operator.

## 3 Discussion

There are many benefits to nuclear decommissioning deployments if embracing increased levels of digital technology, among them improved overall safety and performance. However, challenges remain. For instance, adding technology such as Haptic Digital Twins and Assisted modes of Operation to robotic systems would add complexity to the system, making its management and validation harder. Furthermore, training operators to use such systems would require updating traditional training methodologies to include these new technologies; as HDTs and AO are designed with training in mind, this could potentially reduce entry-level requirements to operate a robotic system. For such sophisticated and complex systems, simplicity of use and having user interfaces that make operations as effortless as possible are essential. This increases the need for research to enable new interaction schemes between highly automated and complex systems, and human operators.

Robotic systems enabling remote operation in normal environments are the most readily available devices in the market, but their operational lifespan can be considerably reduced (Zhang et al., 2020) if used in hazardous environments without further protection. In contrast, bespoke mechanical designs that reduce the number of electronics and plastics in the joints of the robots can improve the operational lifespan of a robot, making them suitable for nuclear decommissioning environments. The use of radiation-proved robotic devices and Commercial Off the Shelf (COTS) robots together with AO and HDT technologies for training and deployment should be a focus for R&D.

Current regulations for most nations like the United Kingdom often require that the operator performing the decommissioning task to be in complete control of any remotely operated vehicle (ROV) or tool during its deployment. By the time assisted and semi-autonomous modes of operations become readily available, such modes would need to include the capability of seamless transition from assisted to manual operation for reconfiguration and recovery in case of failure. Such capability would require an efficient Human Robot Interaction scheme to seamlessly enable transition and ensure optimal task performance.

The introduction of novel digital technologies should be done in such a way to ensure that new concepts or procedures are not detrimental to the success of a deployment, giving time for erroneous ideas to be identified and discarded. Such introduction should include the overall nuclear decommissioning community, including researchers, developers, deployment specialists, regulators, and site owners of different nations. This integrated and paced approach would help developers to build trust in the systems whilst creating a safety case that regulators can validate, and site owners implement.

Considering the state of HDTs and AO, and its rapid adoption rate outside the nuclear industry, we believe the benefits of such technologies will be realized in the coming decade. There is already evidence of nuclear site operators (Sellafield, 2023) and solution providers (UKAEA, 2023; AtkinsRealis, 2024) aligning their priorities around digital technologies and robotics. Increased funding for industry and academia to transition from Low Technology Readiness Level (TRL) to mid and high TRL is underway, with initial prototypes and use case engagement pushing for initial active demonstrators (RAICo, 2024).

## Data Availability

The original contributions presented in the study are included in the article/supplementary material, further inquiries can be directed to the corresponding author.
